# Optimal PSA Threshold for Obtaining MRI-Fusion Biopsy in Biopsy-Naïve Patients

**DOI:** 10.1155/2021/5531511

**Published:** 2021-07-01

**Authors:** Luke L. Wang, Brandon L. Henslee, Peter B. Sam, Chad A. LaGrange, Shawna L. Boyle

**Affiliations:** ^1^Division of Urology, University of Nebraska Medical Center, Omaha, NE 68198, USA; ^2^Division of Urologic Surgery, University of Missouri School of Medicine, Columbia, MO 65212, USA; ^3^Division of Urology, University of New Mexico School of Medicine, Albuquerque, NM 87106, USA

## Abstract

**Objective:**

The study investigates the prostate-specific antigen threshold for adding targeted, software-based, magnetic resonance imaging-ultrasound fusion biopsy during a standard 12-core biopsy in biopsy-naïve patients. It secondarily explores whether the targeted biopsy is necessary in setting of abnormal digital rectal examination.

**Methods:**

260 patients with suspected localized prostate cancer with no prior biopsy underwent prostate magnetic resonance imaging and were found to have Prostate Imaging Reporting and Data System score ≥ 3 lesion(s). All 260 patients underwent standard 12-core biopsy and targeted biopsy during the same session. Clinically significant cancer was Gleason ≥3 + 4.

**Results:**

Percentages of patients with prostate-specific antigen 0–1.99, 2–3.99, 4–4.99, 5–5.99, 6–9.99, and ≥10 were 3.0%, 4.7%, 20.8%, 16.9%, 37.7%, and 16.9%, respectively. Cumulative frequency of clinically significant prostate cancer increased with the addition of targeted biopsy compared with standard biopsy alone across all prostate-specific antigen ranges. The difference in clinically significant cancer detection between targeted plus standard biopsy compared to standard biopsy alone becomes statistically significant at prostate-specific antigen >4.3 (*p*=0.031). At this threshold, combination biopsy detected 20 clinically significant prostate cancers, while standard detected 14 with 88% sensitivity and 20% specificity. Excluding targeted biopsy in setting of a positive digital rectal exam would save 12.3% magnetic resonance imaging and miss 1.8% clinically significant cancers in our cohort.

**Conclusions:**

In biopsy-naïve patients, at prostate-specific antigen >4.3, there is a significant increase in clinically significant prostate cancer detection when targeted biopsy is added to standard biopsy. Obtaining standard biopsy alone in patients with abnormal digital rectal examinations would miss 1.8% clinically significant cancers in our cohort.

## 1. Introduction

While there is increasing utilization of multiparametric magnetic resonance imaging- (mpMRI-) ultrasound fusion biopsy in the detection of localized prostate cancer, this utilization is not uniform and the indications for mpMRI remain imperfectly defined. A recent national survey of urologists and radiation oncologists showed that while both specialties reported positive attitudes towards mpMRI, only about a quarter of providers in both groups actually ordered mpMRI [1]. Likewise, a recent review of SEER data showed that while more people are using mpMRI in men with prior biopsy and no prior biopsy, there remains significant heterogeneity in utilization patterns, with increased utilization reported in patients with higher socioeconomic status [2].

The findings suggest that one of the main factors limiting the accessibility of mpMRI is its affordability [3]. An online marketplace that reports the costs of medical procedures under various insurance plans, New Choice Health, estimates that the national cost of pelvic MRI ranged from $500 to $7,900, with the average being $2,500 [4, 5]. Thus, adding routine mpMRI to the 1–1.2 million prostate biopsies performed annually in the United States would cost approximately 3 billion annually, 15% of the entire cost of managing prostate cancer [5]. Furthermore, it is estimated that approximately only 30% of community hospitals perform mpMRI, with only 25% of hospitals performing more than 20 mpMRI monthly [5, 6]. This draws attention to the fact that most published reports on the diagnostic performance of prostate mpMRI are from tertiary centers with substantial technical expertise [5]. In fact, there is about 54% disagreement in the interpretation of the same prostate mpMRI between community settings and tertiary centers [5, 7]. These challenges to the feasibility of widespread implementation of mpMRI for all patients presenting with suspicion for localized prostate cancer raises the question of whether there is a prostate-specific antigen (PSA) threshold beyond which adding mpMRI significantly increases detection of clinically significant prostate cancer (csPCA). The current study aims to investigate potential PSA thresholds to obtain mpMRI in biopsy-naïve patients presenting with suspected localized prostate cancer.

## 2. Materials and Methods

The retrospective study was approved by the Institutional Review Board at our institution. From 2017 to 2019, patients presenting with suspected localized prostate cancer underwent mpMRI of the prostate as part of routine clinical practice at our institution. Indications for prostate mpMRI in our cohort included concerning PSA or PSA velocity, digital rectal exam (DRE), incidentally detected prostate cancer on transurethral resection of prostate, incidental Prostate Imaging Reporting and Data System (PI-RADS) score ≥3 on MRI obtained for nonurologic indications, or incidental fluorodeoxyglucose positron emission tomography- (PET-) avid lesion of the prostate on PET-scan for nonurologic investigations. Patients who had lesion(s) with PI-RADS version 2.1 score≥3 underwent targeted, software-based, MRI-ultrasound fusion biopsy (targeted biopsy) of all lesions (UroNav system, Philips-Invivo, Gainesville, Florida) followed by 12-core systematic transrectal ultrasound-guided biopsies (standard biopsy) during the same session. Overall, 762 patients underwent mpMRI followed by targeted and standard biopsy during the same session. 260 of these patients had no prior biopsy and thus constituted the biopsy-naïve cohort reported in this study. All biopsies were performed by one of 12 board-certified urologists with the support of dedicated staff who were involved in preparing the setup and intraprocedural assistance. The number of cores taken from each lesion was not standardized due to the retrospective study design. We estimate that at least 2 cores were taken of each lesion with a PI-RADS score ≥3. All internal and external mpMRI were read or overread by one of 6 board-certified radiologists at our institution trained in reading mpMRI of the prostate. All biopsies were reviewed by one of two pathologists. Comparisons within groups were made using the McNemar's test or exact McNemar's test for dichotomous dependent variables, with *p* values <0.05 considered statistically significant. Kendall's tau-b was used to assess the strength of associations between continuous variables and dichotomous variables. All statistical analyses were performed using SPSS version 21 (IBM, Armonk, NY, USA).

## 3. Results

Table 1 describes the baseline characteristics of our cohort. The numbers of patients with PSA 0–1.99, 2–3.99, 4–4.99, 5–5.99, 6–9.99, and 10 and above were 8/260 (3.1%), 12/260 (4.6%), 54/260 (20.8%), 44/260 (16.9%), 98/260 (37.7%), and 44/260 (16.9%), respectively. There were 169/260 (65.0%) csPCA detected in the entire cohort. We assessed the association between prostate volume on mpMRI and the presence of clinically significant cancer on standard biopsy using Kendall's tau-b. There was a statistically significant negative association between prostate volume on mpMRI and the presence of clinically significant cancer on standard biopsy, *τb* = −0.177, *p*=0.001.

**Figure 1:**
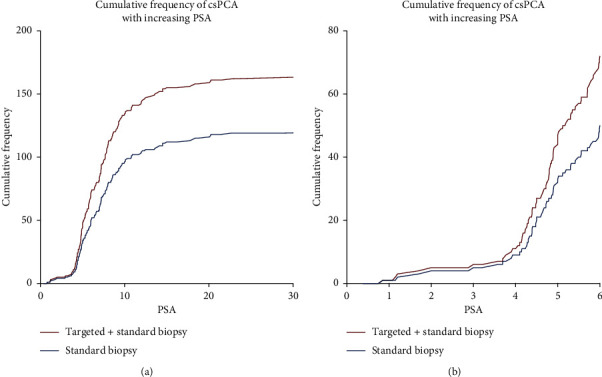
Cumulative frequency of clinically significant prostate cancer with increasing PSA for targeted plus standard biopsy compared to standard biopsy alone. (a) Cumulative frequencies across all PSA ranges reported. (b) Cumulative frequencies across a small PSA range to illustrate the region where the two curves begin to diverge. csPCA = clinically significant prostate cancer; PSA = prostate-specific antigen.

Figures 1(a) and 1(b) show the cumulative frequency of csPCA on targeted plus standard biopsy compared to standard biopsy alone. General inspection suggests that the two curves begin to diverge at PSA of around 4.  Table 2 shows the PSA thresholds when adding targeted to standard biopsy detected more csPCA. As illustrated, the difference in csPCA detection between targeted plus standard biopsy compared to standard biopsy alone becomes statistically significant at PSA >4.3 (*p*=0.031). At this threshold, targeted plus standard biopsy detected 20 csPCA while standard biopsy detected only 14 csPCA. This represents a 43% increase in csPCA detection and saves 38/260 (14.6%) mpMRI.

There were 32 patients who underwent targeted with concurrent standard biopsy with abnormal DRE. Variability in DRE was not assessed due to the retrospective nature of the study design. This limited our ability to discern whether the number of years the physician has practiced was associated with the frequency of DRE. We estimate that the majority of patients had a documented positive or negative DRE prior to biopsy. csPCA was found in 20/32 patients. Of those, 3/32 were found on targeted biopsy only, 2/32 on standard biopsy only, and 15 on both targeted and standard biopsy. Thus, omitting targeted biopsy when there is a positive DRE would save 32/260 (12.3%) mpMRI, miss 3/20 (15.0%) csPCA in the abnormal DRE subgroup, and miss 3/169 (1.8%) csPCA in the entire cohort.

## 4. Discussion

While mpMRI of the prostate is being increasingly adopted, its utilization is complicated by social inequities and inequities in the distribution in healthcare facilities and providers as well as a lack of guidance on when it may be most beneficial [2, 8]. Low-income, rural communities in particular suffer from a lack of access to prostate MRI or from trained professionals available to interpret them [2, 7, 8]. Based on 2010 Census data, approximately 60 million, or 19%, of Americans live in rural areas. If we presume 30% of rural residents had access to prostate MRI and 3% had access to the radiologist who could interpret them based on data from Leake et al. [8], then at least, 42 million people in the United States would not have access to the technology, and 58 million would not have access to specialized radiologists [8]. This is a very conservative estimate as the percentages were based on responses from only 10 community groups and thus subject to nonresponse bias. Furthermore, the population estimate above does not include significant numbers of urban clusters with limited access to mpMRI. Similarly, we postulate that limited access to mpMRI of the prostate and mpMRI-ultrasound fusion biopsies is present globally. For example, in their 2021 report on prostate cancer screening in a single center in Brazil, the authors note that fusion biopsy was not performed due to limited resources [9].

There are now several prospective randomized trials and multicenter studies that suggest that the addition of targeted biopsy in biopsy-naïve patients increases *net* csPCA detection [10–13]. However, these studies do not actually address the question of which individual patient would actually benefit from mpMRI [14]. In other words, they are not designed to answer the question of in what situations *should* a mpMRI be performed to detect an otherwise undiagnosed csPCA on standard biopsy and in what situations can it be omitted because a standard biopsy would have detected it regardless. It is sobering to consider that we currently do not have any novel yet inexpensive risk assessment tools to answer this question. We are left with PSA, DRE, sometimes PSA velocity, and in some cases family history and African American ancestry as the only available tools to stratify risk. In our practice and we postulate more likely than not in most rural practices, the PSA and DRE remain the cornerstone from which to stratify prostate cancer risk.

While, in a resource-rich setting, the inclination and perhaps routine practice may be to obtain mpMRI for all patients with suspicion for localized prostate cancer, this may not be feasible in the setting of technological and resource disparity. Thus, the question arises as to whether one could utilize PSA and digital rectal examination to determine which patients might benefit from mpMRI. The present study shows that mpMRI is unlikely to add diagnostic value in the detection of csPCA at PSA below 4.3. While this is lower than anticipated based on prior published reports, it nonetheless saves 14.6% (38/260) of mpMRIs. Prior to this study, we have been obtaining mpMRI for the majority of patients with concern for localized prostate cancer regardless of PSA, given that mpMRI is readily available and covered by insurance for most patients who seek prostate cancer care at our institution. We are currently evaluating whether mpMRI can be omitted for some patients with PSA less than 4 based on these findings and given that patients with PSA less than 4 are frequently biopsied for abnormal DRE.

We performed a focused literature search on MEDLINE/PREMEDLINE (EBSCOhost interface) and EMBASE for articles defining PSA thresholds, cut-off values, and cut-off points, as well as PSA-based stratification, algorithms, nomograms, decision trees that can define those cases in which MRI-US fusion biopsy was added to standard 12-core biopsy. We found only four reports in the English language literature that specifically addressed PSA thresholds. Choi et al. [14] evaluated csPCA cancer detection rates with standard biopsy compared to targeted biopsy in a propensity score matching analysis [15]. In their propensity-matched cohort (*n* = 222 matched pairs), targeted biopsy detected statistically significantly more csPCA compared to standard biopsy only in patients with PSA 2.5 to <4 [15]. While this would suggest that mpMRI should be obtained at PSA threshold of 2.5, this cutoff was arbitrarily determined. They showed that targeted biopsy detected more csPCA than standard biopsy at PSA 4 to <10 and conversely less csPCA than standard biopsy at PSA 10 to <20, but it showed that these differences were not statistically significant [15]. Interestingly, statistical significance was set at *p* < 0.001 [15].

One abstract reported that targeted biopsy detected statistically significantly more csPCA compared to standard biopsy when PSA is between 4 and 10 and >10 [16]. Similar to the aforementioned study, these thresholds were constructed arbitrarily and do not express a true threshold [16]. Another abstract utilized differences between cumulative frequencies of csPCA on targeted biopsy compared to standard biopsy and determined that at PSA ≥6.15, more csPCA was detected on targeted compared to standard biopsy [17]. The abstract does not specify if the cohort had a history of prior biopsy [17]. Additionally, it is unclear how the 6.15 threshold was determined beyond visual inspection [17]. Furthermore, the abstract compared targeted biopsy alone to standard biopsy alone [17]. The abstract would need to compare targeted plus standard biopsy to standard biopsy alone in order to determine the PSA threshold beyond which adding targeted biopsy would significantly improve csPCA detection.

Shakir et al. [18] investigated the same question in a cohort where the majority of patients underwent prior biopsy and csPCA was defined as Gleason grade group ≥3 [18]. They estimated the PSA threshold to be 5.2 based on the finding that obtaining mpMRI at PSA ≥5.2 with subsequent biopsy would upgrade 90% of standard biopsies to Gleason grade group ≥3 cancers and save 36% of MRIs [18]. However, the reasoning behind choosing the 90% cutoff was not specified. If the cutoff was set at 95%, a lower PSA threshold would result. In addition, comparisons between csPCA detected by targeted biopsy and csPCA detected by standard biopsy at this threshold were not reported [18]. While the results suggest that standard biopsy is unnecessary above this threshold, the article does not report data showing the number of csPCA that would have been missed by targeted biopsy if only standard biopsy was performed above this threshold [18]. Given that most guidelines would caution or dissuade active surveillance for patients with Gleason grade group ≥2, it would seem that csPCA is more commonly accepted as Gleason grade group ≥2 rather than ≥3 [19–22].

Therefore, to the best of our knowledge, the current study is the only study to determine a PSA threshold across the range of PSAs above which adding targeted to standard biopsy statistically significantly increases csPCA detection. Given the limited sample size of our cohort and exclusion of other risk factors such as the size of the prostate on DRE, family history, and African American ancestry, further research is needed in this area to verify the proposed PSA threshold taking into account these factors that are immediately available to the provider at the initial encounter.

Several recent studies suggest using PSA density (PSAD) thresholds to determine when targeted biopsy would be beneficial [23–25]. However, PSAD is usually a function of prostate volume obtained on transrectal ultrasound or mpMRI. Thus, it would not allow us to risk stratify biopsy-naïve patients to determine who would benefit from a mpMRI. While the study utilized mpMRI, there is now increasing evidence that mpMRI exhibits similar diagnostic performance as biparametric MRI [26, 27]. Given these findings, it is possible that our results may be extrapolated to patients receiving biparamatric MRI.

We hypothesized that, in the setting of a positive DRE, targeted biopsy would not enhance csPCA detection because cancer would likely be in the peripheral zone and large enough to be sampled by standard biopsy. The results support this hypothesis by showing that, in the presence of positive DRE, adding mpMRI with subsequent targeted biopsy diagnosed 3 additional csPCA that would have been missed on standard alone. These 3 cancers were all Gleason grade group 2 and there was never upgrading from Gleason grade groups 1–3 to 4-5 when targeted biopsy was added. When evaluating only patients with PSA ≤4.3 and positive DRE, excluding mpMRI would only miss 1 out of the 8 csPCA detected at PSA ≤4.3. Hence, as stated, excluding mpMRI in setting of positive DRE in our cohort of 260 biopsy-naïve patients would save 32/260 (12.3%) MRI and miss 3/169 (1.8%) csPCA in the entire cohort. Of the three csPCA with positive DRE missed by standard biopsy, all had extraprostatic extension (EPE) on final prostatectomy pathology, with PI-RADS ≥3 lesions corresponding to the location of EPE on pathology. One case was biopsy Gleason grade group 2 in a PI-RADS 5 lesion in the peripheral zone. Another case was biopsy Gleason grade group 3 in a lesion in the posterior mid-gland peripheral zone with concurrent biopsy Gleason grade group 2 in two lesions in the mid-gland transitional zone (PI-RADS unable to be classified due to metal artifact limiting diffusion sequences). The third case was biopsy Gleason grade group 2 in a PI-RADS 4 lesion in the anterior fibromuscular stroma. While two of these cases noted a nodule on exam corresponding to the location of EPE on mpMRI and pathology, one case noted a nodule on the exam that did not at all correspond to the location of EPE on mpMRI and pathology. These results draw attention to the finding that a positive DRE does not always lead to prostate cancer diagnosis on biopsy but does increase the positive predictive value of such a diagnosis [28].

The corollary to these observations is that the omission of mpMRI in all cases with abnormal DRE may in turn influence the interpretation and reporting of DRE findings. This may occur when the patient has a subtle nodule. In this scenario, we hypothesize that physicians with a high pretest probability that a patient has a clinically significant cancer could emphasize the nodule and be predisposed to omit mpMRI. In contrast, physicians with lower pretest probability but who still wish to maximize cancer detection may still proceed with mpMRI.

The most recent systematic review and meta-analysis from the Annals of Family Medicine, a collaborative effort of seven family medicine organizations, recommends against digital rectal examination by primary care providers [29]. The United States Preventive Services Task Force also recommends against digital rectal examination in the routine screening of prostate cancer [30]. The current results suggest that the omission of DRE would miss a number of csPCA and has the potential to miss csPCA with adverse features on pathology such as extracapsular extension even in the setting of a low PSA. Further research should be considered to evaluate the role of DRE in the era of mpMRI targeted biopsy of the prostate.

Our study consists of several limitations. It is a retrospective study whereby patients underwent targeted biopsy prior to standard biopsy. While the operator was not blinded to the targeted biopsy results in the retrospective study, the design is likely more consistent with real-world practice. It is more practical to perform targeted biopsy before standard biopsy. This allows the operator to avoid standard biopsy on areas already sampled by targeted biopsy. Furthermore, if the biopsy session is terminated prematurely due to patient discomfort, one would have at least biopsied the PI-RADS lesions using the targeted biopsy. Given that mpMRI-guided targeted biopsy was started at our institution in 2017, the retrospective study design does not account for the learning curves of each individual surgeon. The number of cores taken from each lesion was not standardized due to the retrospective study design. It is furthermore not possible to standardize the mpMRIs as some were obtained outside our institution. Given that all mpMRIs were overread by one of 6 radiologists trained in reading prostate mpMRI, variations in interpretation of PI-RADS scoring may be present.

## 5. Conclusions

The addition of targeted, software-based, mpMRI-ultrasound fusion biopsy significantly increases the detection of csPCA at PSA >4.3 in biopsy-naïve patients. At this threshold, 14.5% mpMRIs could potentially be avoided. Obtaining standard biopsy without mpMRI-guided targeted biopsy when there is an abnormal DRE would avoid 12.3% mpMRI and miss 1.8% csPCA in our cohort.

## Figures and Tables

**Table 1:** Clinicopathologic characteristics of the cohort.

Characteristics-continuous	Mean (SD)	
Age (years)	64.5 (7.4)	
MRI prostate volume (grams)	53.2 (24.5)	

	Median (IQR)	
PSA (ng/mL)	6.5 (4.8–8.6)	

Characteristics-categorical	No.	%
*Race/ethnicity*		
White	239	91.9
Black	13	5.0
Hispanic	3	1.5
Asian/Pacific Islander	1	1.6

*Location of lesions*		
Peripheral	127	48.8
Transitional	62	23.8
Peripheral + transitional	48	18.5
Central	11	4.2
AFS	2	0.8
Peripheral + central	8	3.1
Peripheral + AFS	1	0.4
Transitional + central	1	0.4

*MRI number of lesions*		
1	147	56.5
2	74	28.5
3	31	11.9
4	8	3.1

*Highest PI-RADS score*		
2	1	0.4
3	77	29.6
4	109	41.9
5	73	28.1

*MRI EPE*		
No	224	86.2
Yes	36	13.8

*MRI SVI*		
No	252	96.9
Yes	8	3.1

*Highest Gleason group on fusion*		
0	101	38.8
1	9	3.5
2	77	29.6
3	24	9.2
4	14	5.4
5	35	13.5

*Highest Gleason group on standard*		
0	110	42.3
1	26	10.0
2	61	23.5
3	21	8.1
4	15	5.8
5	27	10.4

SD = standard deviation; IQR = interquartile range; ng = nanograms; mL = milliliter; MRI = magnetic resonance imaging; PSA = prostate-specific antigen; PSAV = prostate-specific antigen velocity; AFS = anterior fibromuscular stroma; PI-RADS = Prostate Imaging Reporting and Data System; EPE = extraprostatic extension; SVI = seminal vesicle invasion.

**Table 2:** PSA thresholds when targeted biopsy plus standard biopsy detected more clinically significant prostate cancer than standard biopsy alone.

PSA threshold (greater than X)	Targeted + standard	Standard	Difference	*P* value	Sensitivity (%)	Specificity (%)
3.5	6	5	1	1.000	95.9	7.7
4.0	11	9	2	0.500	93.5	11.0
4.1	13	10	3	0.250	92.3	13.2
4.2	16	11	5	0.063	90.5	15.4
4.3	20	14	6	0.031	88.2	19.8
4.4	24	18	6	0.031	85.8	22.0
4.5	27	21	6	0.031	84.0	25.3
4.8	36	27	9	0.004	78.7	31.9
5.0	47	33	14	<0.001	72.2	34.1
5.2	51	36	15	<0.001	69.8	40.7
5.5	57	40	17	<0.001	66.3	44.0
6	72	50	22	<0.001	57.4	51.6
7	83	59	24	<0.001	50.9	64.8
10	134	96	38	<0.001	20.7	90.1

PSA = prostate-specific antigen.

## Data Availability

The data used to support the findings of this study are available from the corresponding author upon request.
